# Functions of Heterogeneous Nuclear Ribonucleoproteins in Stem Cell Potency and Differentiation

**DOI:** 10.1155/2013/623978

**Published:** 2013-07-29

**Authors:** Qishan Chen, Min Jin, Jianhua Zhu, Qingzhong Xiao, Li Zhang

**Affiliations:** ^1^Department of Cardiology, The First Affiliated Hospital, School of Medicine, Zhejiang University, 79 Qingchun Road, Hangzhou, Zhejiang 310003, China; ^2^Department of Reproductive Endocrinology, Women's Hospital, Zhejiang University School of Medicine, 1 Xueshi Road, Hangzhou, Zhejiang 310006, China; ^3^Centre for Clinical Pharmacology, William Harvey Research Institute, Barts and the London School of Medicine and Dentistry, Queen Mary University of London, London EC1M 6BQ, UK

## Abstract

Stem cells possess huge importance in developmental biology, disease modelling, cell replacement therapy, and tissue engineering in regenerative medicine because they have the remarkable potential for self-renewal and to differentiate into almost all the cell types in the human body. Elucidation of molecular mechanisms regulating stem cell potency and differentiation is essential and critical for extensive application. Heterogeneous nuclear ribonucleoproteins (hnRNPs) are modular proteins consisting of RNA-binding motifs and auxiliary domains characterized by extensive and divergent functions in nucleic acid metabolism. Multiple roles of hnRNPs in transcriptional and posttranscriptional regulation enable them to be effective gene expression regulators. More recent findings show that hnRNP proteins are crucial factors implicated in maintenance of stem cell self-renewal and pluripotency and cell differentiation. The hnRNPs interact with certain sequences in target gene promoter regions to initiate transcription. In addition, they recognize 3′UTR or 5′UTR of specific gene mRNA forming mRNP complex to regulate mRNA stability and translation. Both of these regulatory pathways lead to modulation of gene expression that is associated with stem cell proliferation, cell cycle control, pluripotency, and committed differentiation.

## 1. Introduction

Stem cells are long-lived biological cells that have remarkable capacity to both self-renew and differentiate into multiple specialized cell types [1]. Different kinds of stem cells including embryonic stem (ES) cells, adult stem/progenitor cells, and induced pluripotent stem (iPS) cells have been explored and discussed over the last decades. ES cells are pluripotent cells derived from the inner cell mass of embryos of blastocyst stage, which can be maintained and expanded indefinitely and possess the capacity to give rise to all cell types of the body [2, 3]. Adult stem cells are found, although scarce, in the most tissues or organs throughout the body after embryonic development. They are able to self-renew during lifetime but become more restricted in terms of potency and self-renewal ability and are called either unipotent or multipotent according to their ability to differentiate into one or several mature cell types, respectively [4]. Adult stem cells usually exist in quiescent state and can be triggered when needed for tissue repair and organ regeneration [5–7]. Discovery and generation of iPS cells from somatic cells such as skin fibroblast is an important breakthrough in stem cell research in recent years. Reprogramming technology using several pluripotency-specific transcription factors, such as combination of OCT4, SOX2, KLF4, and c-MYC [8] or combination of OCT4, SOX2, NANOG, and LIN28 [9], converts somatic cells of the body into stem cells, called iPS cells, which have similar pluripotency to ES cells but possess even more potential in terms of drug screening and discovery, disease modelling, and clinical therapy because of their disease-specific or patient-specific state [10–12]. Recent remarkable progress in stem cell research has brought great optimism and offered the possibility to use them for developmental biology studies, disease modelling, cell replacement therapy, and tissue engineering in regenerative medicine [5, 10–13]. As stem cell research progressing, vast application potential of it in modern and future medicine can be easily deduced. However, before that, clear elucidation of basic molecular mechanisms controlling stem cell biology is of importance.

Stem cell differentiation is the process of transition of specialised cells from undifferentiated cells. Cell types are characterized by different cell morphology and cellular functions which are defined by its specific pattern of gene expression thus, cellular differentiation can be considered as a switch or regulation of gene expression. Although significant progress has been made in understanding of molecular mechanisms of stem cell pluripotency, reprogramming, and lineage specification, it is still insufficient to successfully translate stem cell biology into clinical application. Due to the fundamental and indispensable status of DNA transcription and subsequent posttranscriptional modifications of mRNA in gene expression, one nuclear protein family, heterogeneous nuclear ribonucleoprotein (hnRNP), which is essential in nucleic acids metabolism and function [14, 15], has emerged as a new gene regulatory factor in stem cell potency and differentiation.

The hnRNP proteins are a set of nuclear proteins that bind to nascent RNA polymerase II transcripts to form heterogeneous nuclear RNAs (hnRNA) and that are not stable components of other RNA-ribonucleoprotein complexes [14]. In human cells, there are over 20 major proteins, named hnRNPs A-U, which are the most abundant nuclear proteins in eukaryotes [14, 16]. Earlier, the hnRNPs have been implicated in packaging of nascent pre-mRNAs, a small class of hnRNAs, to prevent degradation and to facilitate subsequent processing [17]. However, in recent years, increasing evidence suggests a diverse function of the hnRNPs in gene regulation ranging from nascent transcript packaging to transcriptional regulation, alternative slicing, nucleocytoplasmic transport, and translational regulation of mRNA, and so forth [16, 18, 19]. Consequently, the hnRNPs seem to be putative regulators of gene expression both at transcriptional and posttranscriptional levels. Unsurprisingly, recent data indicates a crucial role of the hnRNPs in stem cell potency and differentiation.

In the present review, we summarize the general features of the hnRNPs and then discuss the involvement of hnRNPs in stem cell biology and the detailed molecular mechanisms by which hnRNPs facilitate or hinder stem cell differentiation.

## 2. General Structural Features and Functions of hnRNPs

**Figure 1:**
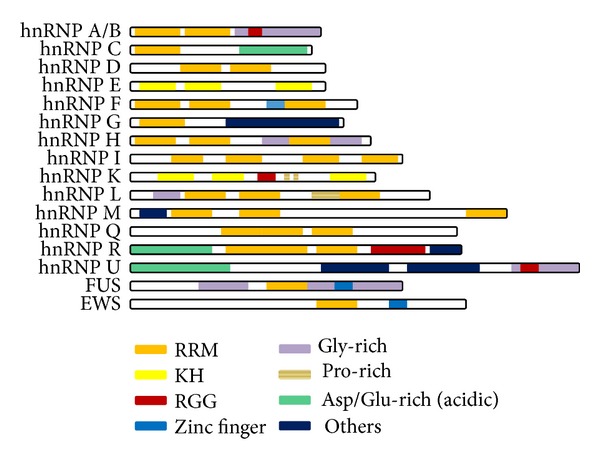
Structure of hnRNPs with multiple modules. Except hnRNP E, K, and U, all other reported hnRNP family proteins contain one or more RRM domains, the structural base responsible for the RNA/ssDNA binding. Instead of RRM domain, hnRNP E and K contain three copies of KH domains. Since KH domain displays relatively weaker RNA/ssDNA binding affinity, it is believed that several copies of KH domains within a given protein are required for achieving greater RNA/ssDNA binding affinity and specificity. RGG repeats domain is the only RNA-binding domain identified in hnRNP U responsible for RNA/ssDNA binding.

Numerous investigations reveal that the hnRNPs are highly divergent groups of proteins with impacts on many aspects of RNA metabolism; however, they share some similar features. The hnRNPs are modular proteins of varying length composed of multiple domains including one or more RNA-binding motifs as well as auxiliary domains (Figure 1). These domains or modules serve as the structural bases of hnRNP functions.

### 2.1. RNA-Binding Motifs in hnRNPs

The most predominant structure of hnRNP family proteins is that all the hnRNPs contain RNA-binding motifs, which mediate general and specific interaction of the proteins with nucleic acids including RNAs and single-strand DNAs (ssDNA). In fact, there are different kinds of RNA-binding motifs in distinct hnRNPs and each hnRNP has one or more RNA-binding modules [14].

The most prevalent and highly conserved RNA-binding motif is RNA recognition motif (RRM), also known as RNP consensus sequence RNA-binding domain (cs-RBD) or RNP motif [20–22]. The RRM is the most extensively studied RNA-binding domain which is approximately 90 amino acids forming a *β*1-*α*1-*β*2-*β*3-*α*2-*β*4 topology as demonstrated by the first and typical RRM [21, 23]. The hall mark of the RRM is the presence of two highly conserved sequences referred to as RNP1 and RNP2, which are separated by about 30 amino acids [22, 24]. RNP1 in the *β*3 strand and RNP2 in the *β*1 strand directly interact with RNA, resulting in the binding of RNA to the *β* sheet surface. In addition, the two external *β* sheets, loops, and C- and N-termini can promote the RNA-binding affinity and facilitate recognition for specific nucleotide sequences [25]. However, the RRM folds into *αβ* structure with some variations. To date, structural analyses have determined more than 30 different RRM structures with unexpected variations. For instance, RRM2 and RRM3 in hnRNP I, known as polypyrimidine tract-binding protein (PTB), have five *β* sheets by inserting an extra *β*5 antiparallel to *β*2 [26, 27]. Moreover, three-dimensional structures of RRMs in complex with nucleic acids in RNA recognition are also versatile which, with the multiformity of RRMs, reflect the notable adaptability of this motif in order to fulfill high affinity and specificity and achieve various functions usually related to posttranscriptional gene regulation [25]. The RRM modules are found in most of the hnRNPs, except for hnRNP K, E, and U (Figure 1), and are necessary and sufficient for RNA binding with high affinity and specificity.

Another RNA-binding motif discovered in hnRNPs is K homology (KH) domain, which is structurally different from the RRM. The KH domain was first identified as nucleic acid recognition motif in hnRNP K protein 20 years ago [28]. In eukaryotes, the type I KH domains are commonly found, which have a *β*1-*α*1-*α*2-*β*2-*β*′-*α*′ structure and interact with RNA or ssDNA though with low micromolar affinity. Therefore, several copies of KH domains within a given protein are required for achieving greater RNA/ssDNA binding affinity and specificity [29]. Among all hnRNPs, the hnRNP K and hnRNP E1/E2, also known as major poly(C)-binding proteins performing a wide range of cellular functions, contain three KH domains that mediate the binding of RNPs to single strand nucleic acids [30] (Figure 1).

RGG domain, which consists of several Arg-Gly-Gly (RGG) repeats interspersed with aromatic residues, is an arginine- and glycine-rich region that is discovered in some hnRNPs [14, 31]. RGG repeats bind with RNA directly or indirectly through association with other RNA-binding motifs [32]. Dimethylation of arginine residues in RGG box is common and represents an important modification in regulating RNA-binding activity [33]. RGG domain is alone or concomitant with other RNA binding modules in distinct hnRNPs. For example, RGG repeats domain is the only RNA-binding domain identified in hnRNP U responsible for nucleic acid binding, while in hnRNP A1 RGG box coexists with RRMs and both of them function as nucleic acid binding domains [14, 31] (Figure 1).

Although there are a variety of other RNA-binding motifs in proteins that bind RNA, such as zinc fingers, arginine cluster, and methionine-rich domains, most of them are not identified in vertebrate hnRNPs [14]. Recent investigation has identified two novel RNA-binding domains in the hnRNP G, carboxyl terminal RNA-binding domain (Cter-RBD) composed of 58 residues in C-terminal region, and nascent transcripts targeting domain (NTD) consisting of residues 186–236 which recognizes RNA and recruits the hnRNP G to nascent transcripts [34]. However, whether these domains are common and conserved in the hnRNP proteins warrants further investigations.

### 2.2. Auxiliary Domains

Auxiliary domains are crucial components of the hnRNPs that collaborate with RNA-binding motifs to exert multiple biological functions. In comparison with RNA-binding motifs, auxiliary domains are more divergent in amino acid sequence and structure, making it difficult to classify. Main auxiliary domains in the hnRNPs include glycine-rich domains, acidic domains, serine-rich portions, and proline-rich regions [15, 35] (Figure 1). The functional significance of auxiliary domains is diverse in different hnRNPs, including strand annealing, protein-protein interaction, and nucleocytoplasmic localization [35].

### 2.3. Posttranscriptional and Posttranslational Modifications

In addition to multiple nucleic acid binding motifs and auxiliary domains, the complexity of hnRNPs is further increased via posttranscriptional and posttranslational modifications. Many paralogues and isoforms of the hnRNPs are generated from alternative splicing of common pre-mRNA. For example, the hnRNP A2 and B1 are identical except for 12 amino acids insertion in B1, probably the products of alternative splicing of the same transcript [36]. However, posttranslational modifications seem more important which modulate the hnRNPs activities during biological processes. Various types of posttranslational modifications have been discovered including phosphorylation of serines and threonines, methylation of arginines, and SUMO modification [14, 15, 37]. The hnRNP A/B, C, K, and U are all phosphorylated in vivo, and the hnRNP A1 and A2 are characterized by methylation of arginines in RGG motifs [14, 38]. The functional implications of these modifications are not clearly defined yet; however, an increasing number of investigations suggest two possible roles. First, they are likely to regulate the binding activity of the hnRNPs to nucleic acids or other proteins and serve as potential controllers of the functions of hnRNPs in cells [39]. Second, posttranslational modifications could be involved in hnRNPs mediated nuclear export or localization [37–39]. 

### 2.4. General Functions of hnRNPs

In general, functions of the hnRNPs in various cellular biological processes are based on their nucleic acid binding properties recognizing a wide range of RNA and ssDNA sequences, along with following formation of nucleotide-protein complexes that mediate ssDNA or RNA processing. The hnRNPs assembling on DNA participate in DNA repair, chromatin remodelling, telomere maintenance, and gene transcription [40–45]. Meanwhile, the hnRNPs interacting with RNA take part in every step of RNA metabolism including mRNA splicing, capping and polyadenylation, trafficking, translation, and turnover [15, 46–48]. Therefore, as crucial factors implicated in gene expression through transcriptional and posttranscriptional regulation, hnRNP proteins are highlighted in many cellular processes, such as tumorigenesis [49]. There are also reports presenting the involvement of hnRNPs in stem cell biology which are discussed in detail below.

## 3. hnRNPs in Maintenance of Stem Cell Self-Renewal and Development Potency

Stem cells maintain their unique self-renewal and development potency properties before they initiate differentiation. hnRNPs have been found to be involved in stem cell proliferation and cell cycle regulation which is vital in stem cell survival and stemness (Table 1).

hnRNP I, more commonly known as polypyrimidine tract-binding protein (PTB/PTBP1), is a multifunctional regulator in RNA splicing and processing and is implicated in internal-ribosome-entry-site-(IRE-S) dependent mRNA translation [50, 64]. Ptb^(−/−)^ ES cells display a severe delay in cell proliferation without aberrant differentiation due to prolonged G2/M phase. Importantly, embryonic lethality has been observed in Ptb^(−/−)^ mice [51] further confirming an important role of PTB in stem cell maintenance and embryonic development. Further studies reveal that PTB interacts directly with IRES region of CDK11(p58), a well-known cell cycle regulator involved in M phase progression [52, 53], to inhibit CDK11(p58) IRES activity and subsequent mRNA translation, resulting in promotion of M phase progression in ES cells [54]. Another study using gene knockout mice reveals that nPTB (PTBP2), the paralogous protein of hnRNP I in nervous system, is expressed in neuronal stem/progenitor cells and is essential for cell survival. Further experiments demonstrate that nPTB regulates neuronal precursor states mainly through inhibiting adult-specific splicing of exons associated with modulation of cell fate, proliferation, and the actin cytoskeleton [55].

hnRNP A/B family, RNA- and DNA-binding proteins extensively modulating transcription, RNA processing, mRNA translation, and telomere biogenesis [43], regulates stem cell self-renewal and maintenance as well. Hrp38, an orthologue of human hnRNPA1, binds to 5′UTR G-rich motif of DE-cadherin gene and initiates IRES-mediated translation of DE-cadherin which promotes anchoring of germline stem cell to its niche and staying undifferentiated. Whereas poly(ADP-ribose) modification of hnRNPs disrupts the interaction of Hrp38 with 5′UTR region of DE-cadherin mRNA and represses its translation [56, 57]. Hrp38 and poly(ADP-ribose) precisely regulate DE-cadherin dependent stem cell maintenance. Moreover, growing evidence indicates that hnRNP A2/B1 is highly expressed in undifferentiated ES cells [58, 59]. Recent evidence demonstrates that expression of hnRNP A2/B1 is essential for maintaining human ES cell epithelial phenotype, self-renewal, and pluripotency [65]. hnRNP A2/B1 knockdown inhibits human ES cell proliferation via repression of G1/S transition which is partially attributed to degradation of cyclin D1, cyclin E, and Cdc25A, and controlled by expression of p27 and phosphorylation of p53 and Chk1 [65].

hnRNP U, also known as scaffold attachment factor A (SAF-A), is able to bind to RNA and DNA to initiate and regulate gene expression transcriptionally [66, 67]. hnRNP U protein is involved in stem cell biology. hnRNP U like-1 protein (hnRNPUL1) is considered as a novel surface molecule marker on undifferentiated human ES cells [60]. In addition, hnRNP U maintains ES cell pluripotency as a modulator of pluripotency factor OCT4 through direct binding to OCT4 proximal promoter and activation of OCT4 gene expression [68].

The last group of hnRNPs found in maintenance of stem cell self-renewal and development potency is RNA binding protein EWS (Ewing sarcoma breakpoint 1, also called EWSR1) and FUS (fused in sarcoma, also called TLS/hnRNP P2). EWS and FUS, also classified into hnRNP family, are two members of FET family of protooncoproteins, consisting of C-terminal RNA-binding domain and N-terminal transcriptional activation domain [69–71]. C-terminal region that contains RRMs, RGG repeats, and zinc finger domain of these two proteins is responsible for their interactions with RNA and ssDNA, while N-terminal has SYGQQS repeats behaving as transcription activator that is essential in transforming activity of oncogenic fusion proteins derived from translocation of EWS or FUS with ETS family of transcription factors such as FLI1 and ERG [61, 72–74]. Endogenous EWS is indispensable for stem cell quiescence and maintenance as depletion of EWS gene promotes early cellular senescence in hematopoietic stem/progenitor cells [62]. EWS regulates stem cell senescence likely via inhibition of p16^INK4a^ expression in stem cells, which is implicated in tumorigenesis of Ewing sarcoma [62, 63]. FUS is required for self-renewal capacity and radioprotection of hematopoietic stem cells since Fus^(−/−)^ hematopoietic stem cells have significantly reduced proliferating and repopulating activity and more susceptible to ionizing radiation due to deficiency in DNA damage repair [75]. However, the underlying molecular mechanism remains unclear and warrants further investigations. Comparing with the understanding of EWS and FUS functions, roles of abnormal chimeric proteins fused by EWS/FUS and ETS family genes that cause Ewing sarcoma are better characterized in stem cell biology. For example, EWS-FLI1 fusion occupies 90% of the cases and has been studied extensively. Expression of EWS-FLI1 blocks bone marrow stem cells to differentiate into adipogenic, osteogenic, or myogenic lineages [76, 77]. Introduction of EWS-FLI1 into bone-marrow-derived mesenchymal stem cells induces its malignant transformation [78, 79]. EWS-FLI1 also regulates expression of miRNA-145 and SOX2 to reprogram mesenchymal stem cells to Ewing sarcoma cancer stem cells [80]. In short, aberrant EWS-FLI1 fusion protein prohibits normal differentiation pathways of mesenchymal stem cells and initiates oncogenic transformation of stem cells. 

## 4. hnRNPs in Smooth Muscle Cell Differentiation from Stem Cells

**Figure 2:**
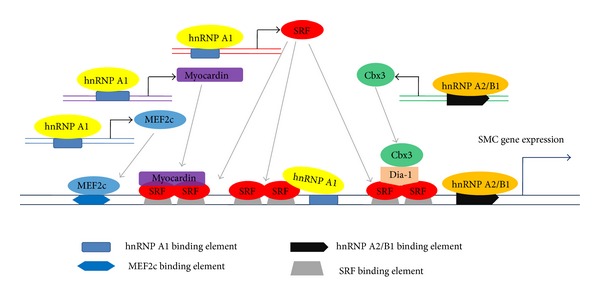
Functions of hnRNP A/B family in SMC differentiation. hnRNP A2/B1 and hnRNP A1 seem to regulate SMC specific gene expression and cell differentiation at two transcriptional levels. hnRNP A2/B1 or hnRNP A1 activates or modulates the transcriptional machinery of SMC specific genes by upregulating another SMC differentiation mediator, Cbx3, or SMC transcription factors and/or co-activators such as SRF, myocardin, and MEF2c, respectively, resulting in SMC differentiation gene expression. hnRNP A1 or hnRNP A2/B1 can also directly regulate SMC differentiation gene expression through SRF binding elements or other specific binding sites within SMC specific gene promoter region.

Smooth muscle cell (SMC) differentiation from stem cell, which is involved in physiological and pathological conditions and regenerative medicine, is a complicated process that involves numerous signaling pathways and molecular interactions. In the past several years, the regulatory networks of gene expression of SMC differentiation have been extensively investigated by our group and others [81–89]. However, until recently, we have discovered and demonstrated that certain hnRNPs of hnRNP A/B family control SMC differentiation from stem cells in vitro and in vivo [90, 91] (Figure 2).

Recent data from our group indicates that hnRNP A2/B1 enhances ES cell differentiation into SMC via transcriptionally modulating SMC specific gene expression through direct binding to promoters of smooth muscle *α*-actin (SM*α*A) and smooth muscle protein 22-*α* (SM22*α*) genes [91]. Furthermore, we demonstrate that chromobox protein homolog gene 3 (CBX3), which is another nuclear protein playing a crucial role in SMC differentiation from stem cells [92], functions as downstream of hnRNP A2/B1 and is required for hnRNP A2/B1 induced SMC differentiation [91]. Taken together, hnRNP A2/B1 promotes SMC differentiation from stem cells both through transcriptional regulation of SMC gene expression and upregulation of Cbx3 expression. Meanwhile, our data also show that hnRNP A2/B1 is essential in embryonic branchial arch artery development, which supports our in vitro findings that hnRNP A2/B1 plays an important role in SMC differentiation [91].

Apart from hnRNP A2/B1, our most recent data also reveals that another hnRNP family member, hnRNP A1, is a key player in regulation of SMC specific differentiation gene expression and SMC development. hnRNP A1 stimulates SMC differentiation from ES cells by two ways: first, it directly binds to promoters of SMC specific genes, SM*α*A gene, and SM22*α* gene and transcriptionally upregulates their expression, for which the binding sites for serum response factor (SRF), a critical transcription factor, within the SMC genes are required and responsible; second, hnRNP A1 regulates SMC specific transcription factors, SRF, myocardin, and myocyte-specific enhancer factor 2C (MEF2c), via transcriptional activation and binding to promoter regions of SRF, MEF2c, and myocardin genes [90].

## 5. hnRNPs in Hematopoietic Stem/Progenitor Cell Differentiation

Differentiation of multipotent hematopoietic stem/progenitor cells into various kinds of blood cell types composes the most important part of hematopoiesis. During differentiation of distinct cell types including erythrocytes and myelocytes, hnRNPs exert posttranscruptional regulations of distinct genes within specific hematopoietic cell lineage. 

### 5.1. hnRNPs in Erythropoiesis

Erythroid precursors undergo enucleation, degradation of mitochondria, and efficient accumulation of hemoglobin to ensure the terminal maturation of erythrocytes.

**Figure 3:**
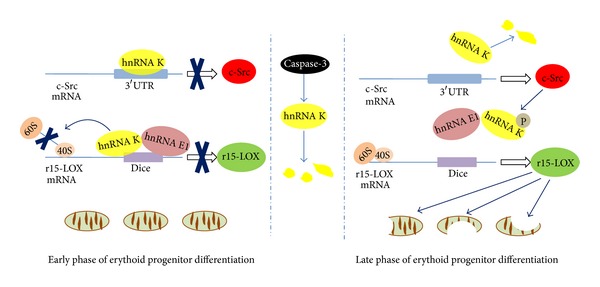
hnRNPs in r15-LOX mediated mitochondria degradation during erythoid differentiation and maturation. hnRNP K functions as a switch for r15-LOX and c-Src gene expression in the early phase of erythoid differentiation, the former is an important mediator regulating erythoid differentiation and erythrocyte maturation and the latter is a tyrosine kinase that phosphorylates hnRNP K activity and forms a feedback loop to regulate r15-LOX gene expression during the late phase of erythoid differentiation or erythrocyte maturation. Importantly, hnRNP K itself is cleaved and inactivated by caspase-3 during erythoid progenitor cell differentiation.

A subgroup of hnRNPs, hnRNP K, and hnRNP E1/E2 which bear three KH domains recognizing CU-rich elements in mRNA 3′UTR function in translational regulation in erythroid differentiation. The breakdown of mitochondria, mediated by reticulocyte-15-lipoxygenase (r15-LOX) which catalyzes mitochondrial membranes, is a key event during erythrocyte differentiation and maturation [93]. The r15-LOX is silenced in early stage of erythroid differentiation but initiated in late step of erythrocyte maturation. In early phase, hnRNP K and hnRNP E1 specifically bind to the differentiation control element (DICE), a repetitive CU-rich sequence, in r15-LOX mRNA 3′UTR region resulting in translational silencing of the gene [94]. This silencing is achieved via the inhibition of 60S ribosomal subunit joining at the translation initiation codon by hnRNP K-E1-DICE complex [95]. In late erythroid differentiation, phosphorylation of hnRNP K by tyrosine kinase c-Src blocks the binding of hnRNP K-E1 to the DICE and leads to activation of r15-LOX mRNA translation and subsequent mitochondria degradation [96]. Interestingly, c-Src, regulator of hnRNP K binding activity, is also controlled by hnRNP K in early stage of the erythroid maturation. hnRNP K directly binds to 3′UTR of c-Src mRNA and inhibits its translation [97]. In addition, evidence shows that caspase-3 is also required for erythroid differentiation [98]. Recent data manifests the cleavage of hnRNP K by caspase-3, which is another way to regulate r15-LOX expression during erythroid cell differentiation [99] (Figure 3).

Accumulation of hemoglobin in differentiating erythroid progenitor cells is a fundamental event in normal erythropoiesis. This process is crucially dependent on stability and translation of *α*- and *β*-globin mRNAs. hnRNP E1/E2 directly interacts with CU-rich sequence in the 3′UTR region of *α*-globin mRNA to form “*α*-complex” that stabilizes the mRNA [100, 101]. The shuttling of hnRNP E1/E2 in the nucleus and the cytoplasm also contributes to mRNA metabolism and gene regulation, such as *α*-globin. Such phenomenon has been nicely described in a set of studies reported by Liebhaber and colleagues [102, 103]. They demonstrate for the first time that hnRNP E1/E2 can load on the nascent transcript of the *α*-globin gene in the nucleus, enhance splicing and nuclear 3′ processing, and then accompany the *α*-globin mRNA to the cytoplasm where it stabilizes the mRNA to extend its functional half-life [102, 103]. More recently, they also demonstrate that hnRNPE1/2 (aCP1/2) plays a pivotal and global role in determining the structure and expression of specific transcripts via its impact on the 3′ processing pathway [104]. 

**Figure 4:**
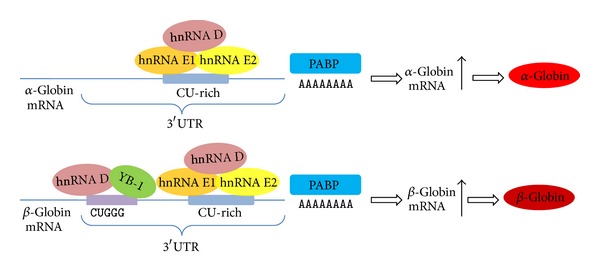
hnRNPs in regulation of hemoglobin expression in erythoid differentiation. Three hnRNP proteins, hnRNP D, E1, and E2, have been suggested to play an important role in the hemoglobin synthesis. All three hnRNP proteins regulate *α*- or *β*-globin mRNA levels through stabilising both mRNAs by directly binding to CU-rich elements within 3′UTR of these genes.

Moreover, hnRNP D, an AU-rich (ARE) binding factor also called AUF1, is identified as a component of *α*-complex [105]. The mRNP complex is positioned in pyrimidine-rich track within the 3′UTR region of *β*-globin mRNA, and the regulatory pathway of *β*-globin gene expression, in which hnRNP E1/E2 has been implicated to play an essential and necessary role, is possibly similar to *α*-globin gene regulation [106]. However, 3′UTR of *β*-globin mRNA harbors several kinds of posttranscriptional regulatory elements [107]. Recent study identifies a novel mRNP *β*-complex composed of hnRNP D and Y box binding protein 1 (YB1), which regulates *β*-globin mRNA stability and sustains high level of *β*-globin mRNA [108]. The mRNP complex comprising hnRNPs mediates erythroid *α*/*β*-globin mRNA stability possibly via facilitating interaction of poly(A) binding protein with mRNA polyadenylate tail, enhancing 3′ processing, and promoting protective effects against its decay [104, 108–110] (Figure 4).

### 5.2. hnRNPs in Myelopoiesis and Myelogenous Leukemia

**Figure 5:**
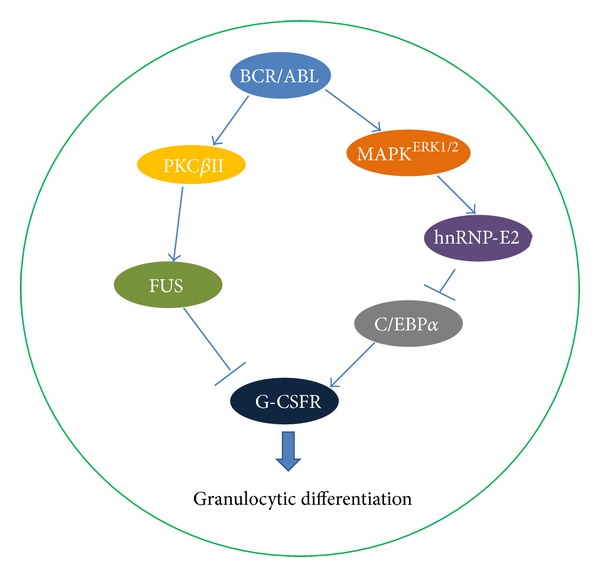
Roles of hnRNPs in the impaired granulocytic differentiation of BCR/ABL transformed myeloid progenitor cells. Both hnRNP E2 and FUS function as the downstream regulators of BCR/ABL oncoprotein and have been implicated in chronic myelogenous leukemia by preventing granulocytic differentiation from myeloid progenitor cells through inhibiting G-CSFR and blocking G-CSF signaling, which finally disrupt the normal myeloid cell differentiation or maturation pathway, resulting in myeloid progenitor cell accumulation abnormally in the bone marrow and circulation.

Myelopoiesis is a process that involves stepwise hematopoietic stem/progenitor cell differentiation. Any disruption or arrest in such differentiation process will result in chronic myelogenous leukemia (CML), a myeloproliferative disorder [111]. BCR/ABL oncoprotein generated by t(9;22)(q34;q11) translocation is responsible for CML induction and progression to fatal blast crisis phase [111]. hnRNPs have been found in normal myelopoiesis and abnormal behaviors of BCR/ABL transformed myeloid progenitors. hnRNP A1 is upregulated in BCR/ABL cells [112]. Shuttling-deficient hnRNP A1 mutant influences survival and granulocytic differentiation of normal myeloid precursors as well as proliferation and tumorigenesis of BCR/ABL transformed myeloid progenitors, suggesting that nucleocytoplasmic shuttling activity of hnRNP A1 is essential and important in the regulation of myeloid progenitor cell differentiation and other functions [112]. FUS is associated with expression of granulocyte-colony stimulating factor receptor (G-CSFR) and G-CSF-stimulated granulocytic differentiation in myeloid precursor cells [113]. FUS expression and binding activity are activated via BCR/ABL regulated PKC*β*II-dependent phosphorylation, preventing granulocytic differentiation and promoting leukemogenesis [113]. Increased level of hnRNP E2 protein is reported in CML myeloid progenitors. hnRNP E2 downmodulates C/EBP*α*, a transcriptional factor crucial for the granulocytic differentiation [114, 115], at translational level through interaction with 5′UTR of C/EBP*α* mRNA [116]. BCR/ABL regulates hnRNP E2 expression depending on enhanced phosphorylation of hnRNP E2 by BCR/ABL-activated MAPK^ERK1/2^, and high level of BCR/ABL is essential to maintain BCR/ABL-MAPK^ERK1/2^-hnRNP-E2-C/EBP*α* differentiation inhibitory pathway in CML myeloid progenitor cells [117] (Figure 5).

## 6. hnRNPs in Differentiation of Neural Stem Cells

Recently, evidence that indicates the involvement of hnRNPs in neural stem cell differentiation is emerging. hnRNP A/B has been postulated to play important roles in differentiation of neural lineage and development of nerve system because of its high and broad expression in mouse developing brains and adult mature brains [118, 119]. Genome-wide quantitative analysis of the gene expression in hnRNP A/B^(−/−)^ mice shows altered gene expression pattern closely related to neural development. Meanwhile, hnRNP A/B^(−/−)^ neural stem/progenitor cells undergo altered differentiation modes, further implying that hnRNP A/B regulates neural stem/progenitor cell differentiation [120]. However, more detailed information and direct evidence of the effects of hnRNPs on neural stem cell differentiation are still lacking and require further investigations.

## 7. Conclusions and Perspectives

Recent findings expand the range of functions of hnRNP proteins far beyond nascent pre-mRNA packaging. They are now viewed as fundamental proteins with diverse roles in almost all the aspects of nucleic acid metabolism from nascent transcripts to mRNA translation. It is not surprising that hnRNPs play significant roles in stem cell maintenance and differentiation due to the key effects of hnRNPs on RNA processing and gene expression. The hnRNPs have been found involved in stem cell self-renewal and potency, smooth muscle cell differentiation, erythropoiesis and myelopoiesis, and neural stem cell differentiation. Uncovered major molecular mechanisms by which hnRNPs regulate stem cell behaviours include transcription initiation through direct binding to promoter sites, mRNA stabilization via forming specific mRNP complex, and mRNA translational regulation by interaction with 3′UTR or 5′UTR region of mRNA, eventually leading to modulation of gene expression which is associated with stem cell proliferation, cell cycle control, and committed differentiation.

Although some achievements have been reached in the field of hnRNPs and stem cells, it is still a long way to comprehensively understand the hnRNP functions and precise underlying mechanisms in stem cell states. Further investigations focusing on hnRNPs in stem cells should be taken to extend the data pools, depict the global regulatory network containing hnRNPs, and uncover utility potential of hnRNPs for medical purposes in stem cells, especially in reprogrammed iPS cells. In fact, study of hnRNPs is difficult owing to their diversified posttranscriptional and posttranslational modifications, dynamic three-dimensional structures, and changes of temporal and spatial distribution. One important work is to crystallize the three-dimensional structures of hnRNPs, their target nucleic acids, and hnRNPs-RNA complexes, which can help to better seize the functional roles of the hnRNPs. Additionally, mutational analysis, genomic database, and bioinformatics approaches can further provide extensive information of functional and structural properties of these biological vital proteins in stem cell maintenance and differentiation.

## Figures and Tables

**Table 1:** hnRNPs in maintenance of stem cell self-renewal and development potency.

hnRNPs	Stem cell types	Functional context	Target gene	RNA/DNA interaction	Reference
hnRNP I/PTB	Embryonic stem cells	Proliferation (G2/M progression)	CDK11(p58)	mRNA translation	[50–53]
hnRNP I/nPTB	Neuronal progenitor cells	Cell survival	(Not identified)	Splicing of exons	[54]
hnRNP A1/Hrp38	Germline stem cells	Staying in niche undifferentiated state	DE-cadherin	mRNA translation	[55, 56]
hnRNP A2/B1	Embryonic stem cells	Proliferation, pluripotency (G1/S transition)	(Not identified)	(Not identified)	[57–59]
hnRNP U/AUF1	Embryonic stem cells	Cell pluripotency	OCT4	Transcription	[60]
EWS	Hematopoietic stem cells	Cell senescence	p16^INK4a^	(Not identified)	[61, 62]
FUS	Hematopoietic stem cells	Cell survival, repopulation	(Not identified)	DNA repair	[63]
